# Prevalence and risk factors associated with the occurrence of *Campylobacter* sp. in children aged 6–24 months in peri-urban Nairobi, Kenya

**DOI:** 10.3389/fpubh.2023.1147180

**Published:** 2023-09-22

**Authors:** Alice Kiarie, Lilly Bebora, George Gitao, Linnet Ochien’g, Noah Okumu, Christine Mutisya, Joseph Wasonga, Sherril Phyllis Masudi, Arshnee Moodley, Maud A. Amon-Tanoh, Julie Watson, Oliver Cumming, Elizabeth A. J. Cook

**Affiliations:** ^1^Animal and Human Health Program, International Livestock Research Institute, Nairobi, Kenya; ^2^Department of Veterinary Pathology, Microbiology and Parasitology, Faculty of Veterinary Medicine, University of Nairobi, Nairobi, Kenya; ^3^Department of Veterinary and Animal Sciences, University of Copenhagen, Copenhagen, Denmark; ^4^Department of Disease Control, London School of Hygiene and Tropical Medicine, London, United Kingdom

**Keywords:** children, infant, *Campylobacter*, diarrhea, LMIC, zoonoses, food safety

## Abstract

**Introduction:**

Campylobacter bacteria is a major cause of foodborne-related bacterial gastroenteritis in humans worldwide. It is known to cause diarrhea in young children which has been shown to directly affect their weight and height as a result of malnutrition. Severe cases of diarrhea can also lead to death. Most of the burden is experienced in resource-limited countries in Africa and Southeast Asia where the disease is linked to poor hygiene and sanitation. The objective of this study was to determine the prevalence of Campylobacter in children aged between 6 and 24 months in Nairobi, Kenya and identify potential risk factors associated with their occurrence.

**Methods:**

A cross-sectional study was carried out between May to December 2021. A total of 585 randomly selected households were visited in two wards (Uthiru/Ruthimitu and Riruta) in Dagoretti South sub-county, Nairobi. A questionnaire regarding how children’s food is handled, the major foods consumed, sanitation and hygiene, and animal ownership was conducted among caregivers to identify associated risk factors. Stool samples were collected from 540/585 children and screened for the presence of Campylobacter using culture-based methods and confirmed through PCR.

**Results:**

Of the 540 children’s stool samples processed, Campylobacter isolates were detected in 4.8% (26/540). Campylobacter jejuni (C. jejuni) was the most common species in 80.8% of positive samples compared to Campylobacter coli (C. coli) in 26.9% of samples. In six samples, both C. jejuni and C. coli were isolated, while in four samples, it was not possible to speciate the Campylobacter. Drinking cow’s milk (OR 4.2, 95% CI 1.4 – 12.6) and the presence of animal feces in the compound (OR 3.4, 95% CI 1.1 – 10.3) were found to be statistically associated with Campylobacter carriage in children.

**Discussion:**

The carriage of Campylobacter in children in this community indicates a need for further investigation on source attribution to understand transmission dynamics and inform where to target interventions. Awareness creation among caregivers on good personal and food hygiene is needed, including boiling milk before consumption. Implementation of biosecurity measures at the household level is highly recommended to reduce contact between animals and humans.

## 1. Introduction

Campylobacteriosis is a major foodborne zoonotic disease, causing gastroenteritis in humans worldwide (1–3). *Campylobacter* infection is hyper-endemic in lower and middle income countries (LMIC) (1). It is estimated that *Campylobacter* organisms are responsible for 100 million foodborne diarrheal cases, and more than 21,000 deaths throughout the world each year (4). The majority (80–90%) of human infections are caused by *Campylobacter jejuni*, with 10–20% of infections caused by *C. coli* (1, 5). Other *Campylobacter* species that have been associated with diarrhea in humans include: *C. lari, C. upsaliensis, C. fetus, C. concisus,* and *C. hyointestinalis* (6).

*Campylobacter* is a leading bacterial cause of diarrhea in children below 2 years of age from high income and LMICs (1, 2, 7). The isolation rate of *Campylobacter* among children below 5 years with diarrhea, from the LMIC’s is estimated to be 5–20%; with majority of the infection seen in children below 2 years (1, 5, 8). This number may be greatly underestimated because of the fastidious nature of *Campylobacter* therefore making it difficult to isolate from stool and due to a lack of sensitive and consistent laboratory diagnosis and surveillance for *Campylobacter* (3). *Campylobacter* is a major cause of bloody diarrhea in children (2), and affects children’s weight gain and linear growth and is therefore associated with stunting in young children (9).

*Campylobacter* are considered commensals in food animals, with poultry being the main reservoir (8, 10). Ingestion of contaminated poultry meat and poor handling practices of raw chicken have been shown to be the main cause of foodborne *Campylobacter* infections in humans (11). Other reported sources of transmission to humans include consumption of contaminated water and raw milk or milk products (8). Presence of animal feces and open garbage in the cooking area, home slaughtering and access to animals in the houses also contribute to the risk of contamination (12).

We conducted a cross sectional study to determine the prevalence of *Campylobacter* in children aged between 6 and 24 months in a low-income peri-urban setting, and to identify potential sociodemographic, hygiene and sanitation as well as biosecurity factors that could be associated with the occurrence of *Campylobacter* in children. The importance of this study is the identification of *Campylobacter* carriage in children 6–24 months. Undetected *Campylobacter* infections may result in malnutrition and exacerbate deficiencies from food insecurity in this resource limited setting. The identification of risk factors is crucial for developing appropriate interventions to reduce infections in children.

## 2. Materials and methods

### 2.1. Study design and study settings

**Figure 1:**
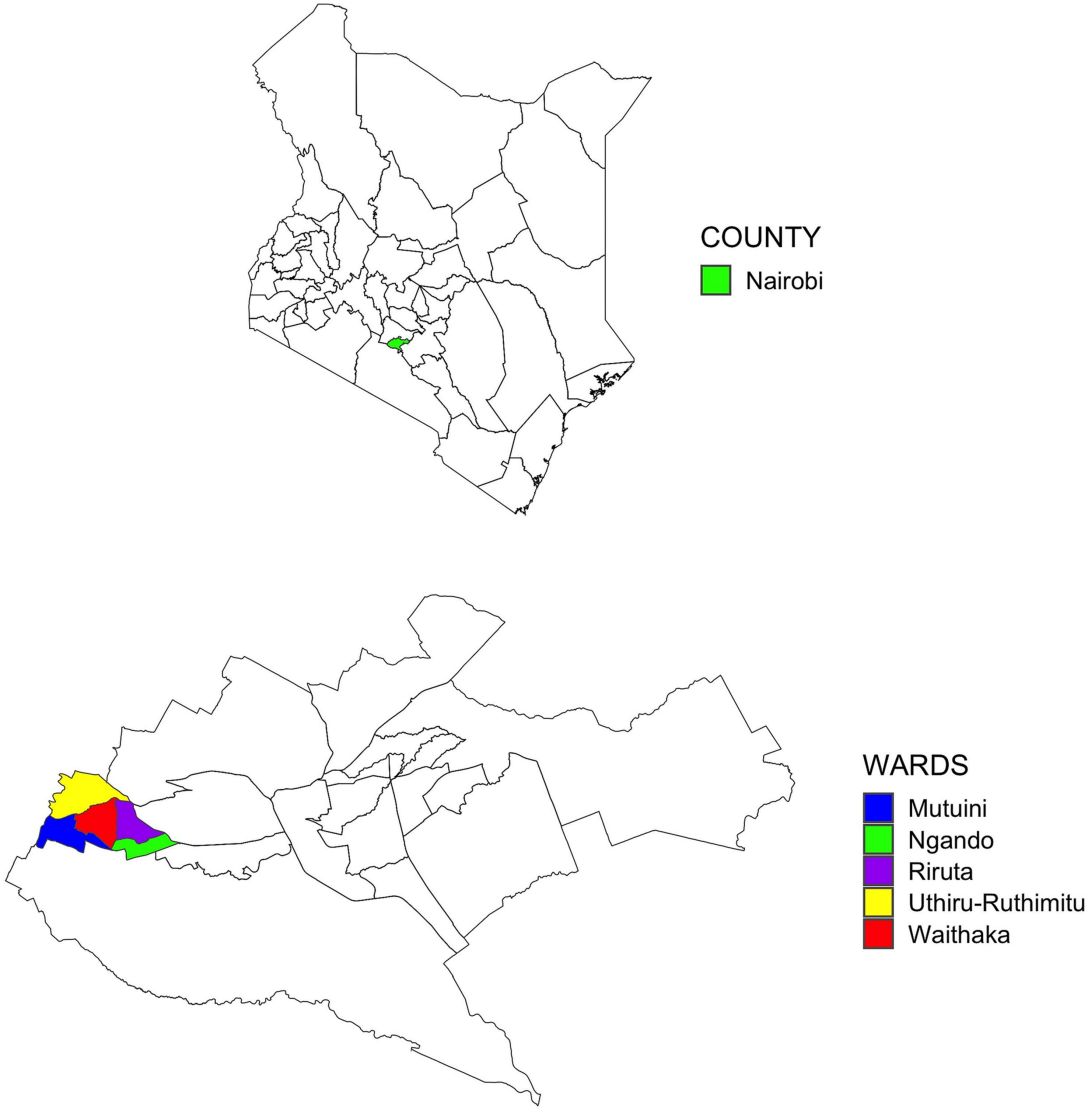
Map of the study area indicating the wards of Dagoretti South Subcounty within Nairobi County (bottom) in Kenya (top).

We conducted a cross-sectional study of households with a child aged between 6 to 24 months from May to December 2021. The study took place in a low-income periurban community in Dagoretti South sub-county, Nairobi, Kenya (Figure 1). As per the 2019 census, Dagoretti is termed a peri-urban area, which comprises over 400,000 people (9.9% of Nairobi’s population) and covers 29.1km^2^ (4.1% of the Nairobi’s surface area) (13). This subcounty has been shown to have poor environmental conditions, a high infectious disease burden, and most dwellers live in low-income settlements (14). The study took place in two wards of the five wards that make up Dagoretti South namely -Uthiru/Ruthimitu and Riruta. Both are classed as low-income settlements (14), with limited water or sanitation infrastructure, poor hygiene, and many residents are livestock keepers or in contact with livestock therefore, at risk of foodborne diseases.

### 2.2. Study population and recruitment strategy

The study population was randomly selected from the two wards. Three community health units (CHUs) were identified, one CHU in Riruta and two CHUs in Uthiru/Ruthimitu. These were selected due to availability of a health centre for referrals when needed. All active Community Health Volunteers (CHVs) were identified in the three CHUs (*n* = 259). Initially, 100 CHVs were randomly selected proportional to the number of CHVs in each community health unit. There were no exclusion criteria. Ninety CHVs were available to participate in the study and provided a list of all eligible households in their catchment area with a child aged between 6 and 24 months. Between five and seven households were randomly selected per CHV to participate in the study, where five was the smallest number of eligible households provided by a CHV. In total, 585 household participated in the study.

### 2.3. Data and sample collection

Nurses were hired as study enumerators and trained in data and sample collection. They visited each household, obtained informed consent from the primary adult caregiver, and conducted a questionnaire survey using Open Data Kit (ODK) Collect App on a Samsung tablet. The structured questionnaire included demographic information on the household size, income, access to services (electricity, water, and sewerage), age of the caregiver and details regarding the child such as sex and age. Information regarding food types eaten by the child in the previous 24 h and any health events were recorded. Information was also collected on water, sanitation, and hygiene conditions, such as the type of drinking water source, the type of sanitation facility, handwashing, and food hygiene practices. Water sources were classified according to the WHO/UNICEF Joint Monitoring Programme for water supply, sanitation and hygiene (JPM) into: safely managed – drinking water from an improved water source that is accessible on premises, available when needed and free from fecal and priority chemical contamination; basic – drinking water from an improved source, provided collection time is not more than 30 min for a roundtrip including queuing; limited – drinking water from an improved source for which collection time exceeds 30 min for a roundtrip including queuing; unimproved – drinking water from an unprotected dug well or unprotected spring; surface water – drinking water directly from a river, dam, lake, pond, stream, canal or irrigation canal. The JPM classification for sanitation include: safely managed – use of improved facilities that are not shared with other households and where excreta are safely disposed of *in situ* or removed and treated offsite; basic – use of improved facilities which are not shared with other households; limited – use of improved facilities shared between two or more households; unimproved – use of pit latrines without a slab or platform, hanging latrines or bucket latrines; open defecation – disposal of human feces in fields, forests, bushes, open bodies of water, beaches, and other open spaces or with solid waste (15).

From each recruited child, a stool sample was collected in a disposable diaper (Pampers, Proctor and Gamble, Cincinatti, Ohio, United States). The caregiver was instructed on how to fit the diaper and remove it after the child defecated. A sealable plastic bag labelled with the household ID and date was also supplied and the diaper with the sample was placed inside. Repeated visits were made to caregivers to collect stool up to a week following the original visit. Diapers were collected from the household upon caregiver’s notification to the CHV within 2 h, placed in a cool box, maintained at under 4°C with ice packs and delivered to the International Livestock Research Institute (ILRI) laboratories to be processed within 6 h.

### 2.4. Isolation and identification of *Campylobacter*

Stool samples were prepared for enrichment and cultured according to the protocol by Global Salm-Surv (16). Initially, 0.2 grams of feces was enriched in Preston broth and incubated at 38° C overnight under microaerophilic conditions generated using CampyGen Gas Generation Sachets (Oxoid, Thermo Fischer Scientific, Basingstoke, Hampshire, United Kingdom). After 24 h, 10 μL of each of the incubated samples was streaked onto modified Charcoal Cefoperazone Deoxycholate agar (mCCDA) (Oxoid, Thermo Fischer Scientific, Basingstoke, Hampshire, United Kingdom) plates and incubated at 38° C for 48 h under microaerophilic conditions. Plates were examined for presence of presumptive *Campylobacter* colonies by morphological characteristics (17). A suspect colony was Gram-stained and viewed under a light microscope at x100 magnification lens under oil emersion (16). Gram negative curved rods, some forming s-shapes, were sub-cultured onto a Columbia blood agar plate and incubated under micro-aerophilic conditions for 24 h at 38° C. Further characterization of the isolates was done by catalase and oxidase tests (16). Differentiation between *C. jejuni* and *C. coli* was done using the hippurate hydrolysis test.

DNA was extracted from suspected *Campylobacter* colonies grown on 5% sheep blood agar for 24 to 48 h at 38°C under microaerophilic conditions by boiling (18). Suspect isolates were confirmed to be of *Campylobacter* genus and *C. jejuni* or *C. coli* species by PCR (18, 19). The primer sequences and the respective PCR conditions are outlined in Supplementary Table S1.

### 2.5. Ethical approvals and participant consent

Before data collection, ethical approvals were sought from the London School of Hygiene and Tropical Medicine (LSHTM) Observational / Interventions Research Ethics Committee Reference 17,188 and the International Livestock Research Institute—Institutional Research Ethics Committee (ILRI-IREC) with project reference ILRI-IREC2019-26. ILRI-IREC is accredited by the National Commission for Science, Technology and Innovation (NACOSTI) in Kenya. Project and individual student approval was also acquired from NACOSTI. The study was conducted in accordance with the Declaration of Helsinki and each caregiver gave signed informed consent. Samples were collected according to the institutional guidelines and in accordance with the approved protocol.

### 2.6. Data cleaning

The data was assessed visually and programmatically to identify any quality and tidiness issues. Variables with too many categories were regrouped for ease of analyses. Missing values were assessed and addressed. Using R version 4.2.1, each household metadata was linked to corresponding samples and laboratory results inconsistences were discussed between the field and laboratory team to clean the dataset.

### 2.7. Data analysis

Descriptive summary statistics were calculated to describe prevalence and general household characteristics. A two-step statistical logistic regression analysis, using univariable and a backward stepwise multivariable approach, was performed to identify risk factors associated with the occurrence of *Campylobacter* in children. The *lme4* package in R statistical software (version 4.2.2) was used to build multilevel logistic regression models (20). The CHV was used as a random effect to account for clustering in the model regarding risk factors for *Campylobacter* in children. For univariable analysis, all variables with a *p*-value lower than 0.2 were retained for assessment in the multivariable analyses. For multivariable analysis, a backward stepwise approach was utilized to select the final logistic regression model using the variables identified from the univariable analysis to identify risk factors responsible for the occurrence of *Campylobacter* in children. The CHU was included as a fixed effect to account for any variation between units. Odds ratio (OR) and 95% confidence intervals (CI) were calculated and *p*-values less than 0.05 were considered statistically significant. Two-way interactions between predictors were assessed using a likelihood ratio test and considered significant if *p* ≤ 0.05. To evaluate for collinearity effect between predictors, the level of association between risk factors identified during the univariable analysis were assessed using a Fisher’s test, and risk factors with more than two-fold changes in the logistic regression coefficients were also checked during the selection process.

The final model was considered valid if the residuals plot versus fitted values plot for each fixed effect showed no clear clustering patterns nor outliers, and deviations from the empirical and expected quantile distribution were not significant (*p*-value > 0.05) using the *simulateResiduals* function from the *DHARMa* package (21). A Q-Q plot was visualised to detect deviations from the expected distribution which included goodness-of-fit tests such as tests for correct distribution, overdispersion and outliers. Variance Inflation Factors (VIFS) were calculated to check for collinearity. VIFS >4 were considered a problem and the variable removed from the model.

## 3. Results

### 3.1. Descriptive statistics of the household characteristics

We recruited 585 households into the study from May to October 2021. The mean household size was four people (range 2–14). Most people lived in a rented house (463/585, 79.1%), used electricity as a source of lighting (583/585, 99.6%), and the houses had cemented floors (564/585, 96.4%). The preparation of child food was predominantly done inside the house (578/585, 98.8%) and preparation surface was always cleaned beforehand. More than half of the households did not have a hand washing facility (321/585, 54.9%). Majority of the recruited households sourced their drinking (513/585, 87.7%) and cooking water (505/585, 86.3%) from basic sources according to WHO/UNICEF Joint Monitoring programme (JPM). Only half of the households indicated treating drinking water (277/585, 47.4%) before use. Treatment of cooking water was not a common practice in the households (77/585, 13.2%). Sanitation facilities were mainly basic (354/585, 60.5%), limited (117/585, 20%) and unimproved (74/585, 12.6%).

Almost all caregivers had at least primary school education (581/585, 99.3%) and most had secondary education (428/585, 73.3%). Household income was mostly non-permanent with casual labour (270/585, 46.2%) or petty trading (164/585, 28.4%) being the most common sources of income. Of the recruited households, 167/585 (28.5%) indicated they owned animals and 436/585 (74.5%) indicated to be in contact with animals in the compound.

We recruited 585 children aged 6–24 months into the study. The children were equally divided between male (*n* = 293) and female (*n* = 292). The number of participants in each age category was also evenly distributed (6–11 months *n* = 187; 12–17 months *n* = 204; 18–23 months *n* = 194). Almost one fifth of children were reported to have had diarrhea in the past week (including the day of sampling prior to the interview) (113/585, 19.3%).

### 3.2. *Campylobacter* prevalence

Stool samples were collected from 540/585 children. Caregivers of the 45 children who did not give a sample either refused or were unable to provide a sample. *Campylobacter* was detected in 26/540 stool samples giving a prevalence of 4.8% (95% CI 3.3 – 7.0%). *C. jejuni* (*n* = 21) was the most frequently isolated and accounted for 80.8% of positive samples compared to *C. coli* which was detected in 7 (26.9%) of positive samples. Six children had co-carriage with *C. jejuni* and *C. coli* at a prevalence of 1.1% (95% CI 0.5 – 2.4%) or 6/26 (23.1%) positive samples. There were 4 isolates 0.7% (95% CI 0.3 – 1.9%) that were *Campylobacter* species, but were not identified as belonging to either *C. jejuni* or *C. coli*, which accounted for 15.4% of positive samples (Table 1).

**Table 1:** Prevalence of *Campylobacter* species detected in children aged 6–24 months in Dagoretti South Subcounty, Nairobi.

*Campylobacter* species	Number of children affected (*n* = 540)	Prevalence (95% CI)
*C. jejuni* (single infection)	15	2.8% (95% CI 1.7 – 4.6%)
*C. coli* (single infection)	1	0.2% (95% CI 0 – 1.0%)
Mixed infections *C. jejuni and C. coli*	6	1.1% (95% CI 0.5 – 2.4%)
Not identified	4	0.7% (95% CI 0.3 – 1.9%)
Total	26	4.8% (95% CI 3.3 – 7.0%)

### 3.3. Risk factor analysis for *Campylobacter* carriage in children

The univariable analysis for risk factors for *Campylobacter* occurrence in children included age, gender, livestock ownership, contact with livestock, food consumed, and source of water (Table 2). Chicken ownership (OR 3.0, 95% CI 1.3 – 7.1), goat ownership (OR 5.4, 95%, 1.2 – 24.3), drinking cow’s milk (OR 3.9, 95% CI 1.3 – 11.8) and presence of animal feces in the compound (OR 4.4, 95% CI 1.4 – 14.3) were significantly associated with *Campylobacter* carriage in children. There was also a significant difference in *Campylobacter* in children between CHUs, with children from Ruthimitu more likely to be positive (OR 7.1 95% CI 1.6 – 30.8). There was no association between *Campylobacter* prevalence and a recent episode of diarrhea (within the previous 7 days including the day of sampling) (OR 0.8, 95% CI 0.3 – 2.5) (Supplementary Table S2).

**Table 2:** Results of univariable analysis of variables measured for association with *Campylobacter* positivity in children from Dagoretti South Subcounty, Nairobi and considered for inclusion in the multivariable model.

Characteristic	Total surveyed (*n* = 585)	Total samples (*n* = 540)	*Campylobacter* positive (%)	Odds ratio (95% CI)	*p*-value
Community health unit					
Riruta	161	145	2	1	
Ruthimitu	239	202	20	7.1 (1.6 – 30.8)	0.009*
Uthiru	185	169	4	1.7 (0.3 – 9.4)	0.547
Hygiene practices					
Drinking water treated	277	255	9 (3.5)	0.6 (0.2 – 1.3)	0.190
Drinking water not treated	308	285	17 (6.0)	1	
Animal feces in the compound	42	36	5 (13.9)	4.4 (1.4 – 14.3)	0.013*
No animal feces in the compound	543	504	21 (4.2)	1	
Garbage in compound	197	183	12 (6.6)	1.9 (0.8 – 4.5)	0.165
No garbage in compound	388	357	14 (3.9)	1	
Food consumption					
Consumed cow’s milk	350	324	22 (6.8)	3.9 (1.3 – 11.8)	0.016*
Did not consume cow’s milk	235	216	6 (2.8)	1	
Animal ownership					
Chicken ownership Yes	101	95	10 (10.5)	3.0 (1.3 – 7.1)	0.013*
Chicken ownership No	484	445	16 (3.6)	1	
Goat ownership Yes	14	14	3 (21.4)	5.4 (1.2 – 24.3)	0.029*
Goat ownership No	571	526	23 (4.4)	1	
Dog ownership Yes	36	34	4 (11.8)	2.6 (0.8 – 8.8)	0.132
Dog ownership No	549	506	22 (4.3)	1	

* *p*-value < 0.05.

### 3.4. Multivariable mixed effects logistic regression model for *Campylobacter* carriage

The results of the final multivariable mixed effect logistic regression model are presented in Table 3 with the backwards stepwise approach presented in Table 4. In the final model, we included two effects (drinking milk, and animal feces in the compound), CHU was included as a fixed effect to account for any variation between units, and a random effect (CHV) was incorporated for the dependency among observations.

**Table 3:** Results of multivariable analysis for *Campylobacter* carriage in children from Dagoretti South Subcounty, Nairobi.

Characteristic	Odds ratio (95% CI)	*p*-value	Variance inflation factors (VIFS)
Animal feces in the compound	3.4 (1.1 – 10.3)	0.028	1.004
No animal feces in the compound	1		
Consumed cow’s milk	4.2 (1.4 – 12.6)	0.010	1.006
Did not consume cow’s milk	1		

**Table 4:** Comparison of mixed-effects logistic regression risk factor models for *Campylobacter* carriage in children.

Model	AIC
Campylobacter positive ~ CHU + Drink cow’s milk+ Feces in the compound+ Owning goats + Owning chickens + garbage in compound + dog ownership + (1|CHV_ID)	197.6
Campylobacter positive ~ CHU + Drink cow’s milk+ Feces in the compound+ Owning goats + Owning chickens + garbage in compound + (1|CHV_ID)	195.8
Campylobacter positive ~ CHU + Drink cow’s milk+ Feces in the compound+ Owning goats + Owning chickens + (1|CHV_ID)	194.4
Campylobacter positive ~ CHU + Drink cow’s milk+ Feces in the compound+ Owning goats + (1|CHV_ID)	193.2
Campylobacter positive ~ CHU + Drink cow’s milk+ Feces in the compound+ (1|CHV_ID)	193.1

For each model, the model formula with the Akaike Information Criterion (AIC) values are provided. CHU, community health unit; CHV_ID, community health volunteer identity.

The final model had the lowest Akaike Information Criterion (AIC) of 193.1. Model validation showed no obvious clustering patterns of simulated residuals nor over dispersion, zero-inflation and outliers tests were not significant (*p*-value > 0.05).

Risk factors identified in the final model to be significantly associated with *Campylobacter* carriage in children included: drinking cows milk (OR 4.2, 95% CI 1.4–12.6), and presence of animal feces in the compound (OR 3.4, 95% CI 1.1 – 10.3) (Table 3).

## 4. Discussion

This study determined the prevalence of *Campylobacter* in children and identified risk factors associated with *Campylobacter* carriage in children in Dagoretti South, Kenya. The prevalence of *Campylobacter* carriage was 4.8%. The prevalence among these children is similar to what has been reported in other African countries. In children below 5 years old, *Campylobacter* prevalence has been shown to range between 2% in Sudan and 21% in South Africa (22). The majority of *Campylobacter* positive children were asymptomatic (76.9%). This is consistent with a recent case control study in children under 5 years in Malawi which showed no difference in *Campylobacter* prevalence in children with or without diarrhea (23). In contrast, a study conducted in Morogoro, Tanzania, indicated a higher prevalence of *Campylobacter* in symptomatic (12.9%) compared to asymptomatic individuals (6.7%) (24). In South Africa, a similar finding was reported where the prevalence of *Campylobacter* was higher among diarrheal stool samples (20.4%) compared to non-diarrheal stool samples (12.4%) (5). Asymptomatic occurrence of *Campylobacter* could be a consequence of a resolved infection since the bacteria may be excreted in the feces up to 12 to 40 weeks after infection (25). Immunity may develop from continued exposure to low doses due to poor hygienic/sanitary conditions and contact with reservoir animals such as chickens (25).

*C. jejuni* was the most common species (80.8%) followed by *C. coli* (26.9%) in our study. Previous studies have identified *C. jejuni* as the leading bacterial cause of food-borne diseases in both high income and lower and middle income countries, causing human bacterial gastroenteritis (5, 24). Our results are similar to another study conducted in Western Kenya, where *C. jejuni* in children was identified in 89.2% and *C. coli* in 10.8% of isolates (8), and consistent with a recent global review which showed similar findings with respect *to C. jejuni* and *C. coli* prevalence (26).

Our study detected co-carriage of *C. jejuni* and *C. coli* in 23.1% of the positive samples. Coinfections were mainly detected among samples which showed swarming growth characteristics during culture, and it was therefore difficult to pick a single colony for subculturing. Co-infections have been described in other studies (27). Co-occurrence of four *Campylobacter* species in stool from asymptomatic children aged below 2 years was also recorded in children from Ethiopia (28). The co-occurrence suggests that *Campylobacter* colonization of children occurred through multiple reservoirs or from a reservoir in which several *Campylobacter* species co-inhabited.

Other uncharacterized species of *Campylobacter* were identified in 15.4% of isolates. Occurrence of other *Campylobacter* species including: *C. upsaliensis*, *C. hyointestinalis*, *C. lari,* and *C. fetus* have been identified from children and animal stool (28, 29). A study involving asymptomatic school going children in Kibera, Kenya, also identified *C. lari* in 1.9% of children (30).

*Campylobacter* organisms were initially identified using culture methods and then confirmed using genus and species level primers (18). This process was laborious and due to the nature of the testing algorithm mixed infections with other unidentified *Campylobacter* species might not have been detected. This was particularly true for samples that were positive for *C. jejuni* and *C. coli* as these were not further screened for other species, and this may have underestimated the carriage of additional *Campylobacter* organisms.

Direct PCR on fecal material without enrichment has been described with comparable sensitivity and specificity to conventional culture techniques (31). However, this earlier work was done on clinical samples where the number of organisms was likely higher, and this approach may not have been effective in our population which was mostly asymptomatic (32). In future the use of multiplex PCR methods may reduce the time required to screen samples for multiple pathogens without impacting sensitivity or specificity (33, 34).

Risk factors identified in the multivariable model to be significantly associated with *Campylobacter* carriage in children included: drinking cow’s milk (OR 4.2, 95% CI 1.4–12.6), and presence of animal feces in the compound (OR 3.4, 95% CI 1.1-10.3). In previous studies, consumption of raw milk was linked with *Campylobacter* outbreaks (8, 35). All milk types available in Kenya, unpackaged, fresh pasteurized, and ultra-high temperature (UHT), may be contaminated and bacterial contamination of milk can occur from the point of purchase to consumption (36). Interventions for improving food hygiene and to reduce contamination of complementary foods for weaning children may target caregiver practices such as boiling milk, treating water, handwashing with soap, and food storage practices (37). However these practices are likely to be determined by the social and economic status of the caregiver so recommendations need to be context specific (37–39).

There are economic, nutritional and sociocultural reasons for keeping animals in peri-urban areas and animals are commonly kept within the living areas (40). In the households that were visited, animals were housed in structures attached to the main house and were free to roam during the day and only housed during the night. The free-range farming, especially of chickens, coupled with low biosecurity and poor hygiene contributed to the presence of animal feces in the compound. Children were noted to be playing in the same compound therefore increasing contact with the feces. Animal ownership and animal waste in the compound is associated with contamination of household drinking water (41). *Campylobacter* organisms are frequently isolated from animal feces, especially livestock (poultry, cattle, sheep, goat, and swine) and pets (3). Shedding of *Campylobacter* organisms by livestock into water sources, environment, and food can cause infection in children through poor personal hygiene practices such as not washing hands regularly, unhygienic food handling practices and poor water hygiene practices (35). Interventions to reduce zoonotic disease transmission in periurban areas should focus on sanitation and biosecurity measures, free-range farming should be discouraged, animals should not be in the same house as humans, and manure should not be used as fuel.

## 5. Conclusion

This study provides evidence of asymptomatic and symptomatic *Campylobacter* infections in children, with *C. jejuni* being the most common species. In future studies, it would be important to understand the incidence and maintenance of *Campylobacter* infection in this age group and to understand the impact on malnutrition particularly in settings with food and nutritional insecurity. Future work may also consider direct molecular approaches for detection of pathogens to reduce time and resources spent in the laboratory and generate results in real-time.

Drinking cow’s milk and presence of animal fecal material within the compound of the household were associated with carriage of *Campylobacter* in children. As well as the potential transmission of *Campylobacter* from animals to children, the occurrence of *Campylobacter* in asymptomatic children may pose a threat to public health as the organisms are continuously shed in feces which can promote spread of the disease to other humans and or animals. Further research work using genotypic methods for determination of relatedness of the isolates circulating in animals, humans and the environment is required for source attribution of the isolates in order to target intervention measures to the most incriminated source.

A “One Health” approach is recommended where multisectoral collaborative efforts from veterinary, human medicine, and environmental managers is required to design efficient and effective strategies for prevention and control of infections both in human and animal populations and reduce contamination of food and the environment.

## Data availability statement

The raw data supporting the conclusions of this article will be made available by the authors, without undue reservation.

## Ethics statement

The studies involving humans were approved by the London School of Hygiene and Tropical Medicine (LSHTM) Observational/Interventions Research Ethics Committee Reference 17188 and the International Livestock Research Institute—Institutional Research Ethics Committee (ILRI- IREC) with project reference ILRI-IREC2019-26. ILRI-IREC is accredited by the National Commission for Science, Technology and Innovation (NACOSTI) in Kenya. The studies were conducted in accordance with the local legislation and institutional requirements. Written informed consent for participation in this study was provided by the participants’ legal guardians/next of kin.

## Author contributions

AK and EC: concept, methodology, data collection, analysis, and manuscript preparation. LB and GG: supervision. LO and MA: methodology and analysis. NO: data collection and analysis. CM and JW: analysis. SM: data collection. AM-T: supervision and funding. JW: concept and methodology. OC: concept, methodology, and funding. All authors contributed to the article and approved the submitted version.
