# Supplementary material for: Prevalence and risk factors associated with the occurrence of Campylobacter sp. in children aged 6–24 months in peri-urban Nairobi, Kenya
Source: Front Public Health. 2023 Sep 22;11:1147180. doi: 10.3389/fpubh.2023.1147180 (PMC10556691; doi:10.3389/fpubh.2023.1147180)
Supplement: Supplementary file 1 [file Table_1.docx]

**Supplementary Table 1: Primer sequences with annealing temperatures and product size for *Campylobacter* genus and species.**

| Bacteria | Gene | Primer | Sequence 5’ to 3’ | AnnealingTemp °C | Product Size (bp) | Ref |
| --- | --- | --- | --- | --- | --- | --- |
| *Campylobacter* | 16S rRNA | MD16S1-F | ATCTAATGGCTTAACCATTAAAC | 59 | 857 | (Müller et al. 2011; Adiguzel et al. 2018) |
|  |  | MD16S1-R | GGACGGTAACTAGTTTAGTATT |  |  |  |
| *C. jejuni* | mapA | MDmapA1-F | CTATTTTATTTTTGAGTGCTTGTG | 59 | 589 |  |
|  |  | MDmapA1-R | GCTTTATTTGCCATTTGTTTTATTA |  |  |  |
| *C. coli* | cueE | MDcol-F | AATTGAAAATTGCTCCAACTATG | 59 | 462 |  |
|  |  | MDcol-R | TGATTTTATTATTTGTAGCAGCG |  |  |  |

Adiguzel MC, Sigirci BD, Celik B, Kahraman BB, Metiner K, Ikiz S, et al. Phenotypic and genotypic examination of antimicrobial resistance in thermophilic *Campylobacter* species isolated from poultry in Turkey. Journal of Veterinary Research (Poland). 2018;62(4):463–8.

Müller W, Böhland C, Methner U. Detection and genotypic differentiation of Campylobacter jejuni and Campylobacter coli strains from laying hens by multiplex PCR and fla-typing. Research in veterinary science. 2011 Feb 1;91:e48-52.
