# Supplementary material for: Prevalence and risk factors associated with the occurrence of Campylobacter sp. in children aged 6–24 months in peri-urban Nairobi, Kenya
Source: Front Public Health. 2023 Sep 22;11:1147180. doi: 10.3389/fpubh.2023.1147180 (PMC10556691; doi:10.3389/fpubh.2023.1147180)
Supplement: Supplementary file 2 [file Table_2.docx]

**Supplementary Table 2 Univariable analysis of all variables measured for association with *Campylobacter* positivity in children from Dagoretti South Subcounty, Nairobi**

| **Characteristic** | **Total**  **Surveyed**  **(n=585)** | **Total**  **Samples**  **(n=540)** | ***Campylobacter***  **positive (%)** | **Odds ratio**  **(95% CI)** | **p-value** |
| --- | --- | --- | --- | --- | --- |
| **Community health unit** |  |  |  |  |  |
| Riruta | 161 | 145 | 2 | 1 |  |
| Ruthimitu | 239 | 202 | 20 | 7.1 (1.6 – 30.8) | 0.009* |
| Uthiru | 185 | 169 | 4 | 1.7 (0.3 – 9.4) | 0.547 |
| **Child characteristics** |  |  |  |  |  |
| Female | 292 | 266 | 14 (5.3) | 1 | 0.493 |
| Male | 293 | 274 | 12 (4.4) | 0.7 (0.3 – 1.7) |  |
| **Age groups** |  |  |  |  |  |
| 6 – 11 months | 187 | 170 | 9 (5.3) | 1 |  |
| 12 – 18 months | 204 | 188 | 9 (4.8) | 0.9 (0.3 – 2.4) | 0.819 |
| 19 – 24 months | 194 | 182 | 8 (4.4) | 0.9 (0.3 – 2.4) | 0.762 |
| Child had fever | 127 | 117 | 6 (5.1) | 1 (0.39 – 2.8) | 0.927 |
| Child did not have fever | 475 | 422 | 20 (4.7) | 1 |  |
| Diarrhea in the  preceding 7 days | 113 | 104 | 4 (3.8) | 0.8 (0.3 – 2.5) | 0.71 |
| No diarrhea in the preceding 7 days | 470 | 434 | 22 (5.1) | 1 |  |
| **Hygiene Practices** |  |  |  |  |  |
| Private toilet | 451 | 418 | 21 (5.0) | 0.7 (0.2 – 2.0) | 0.476 |
| Shared toilet | 134 | 122 | 5 (4.1) | 1 |  |
| Fixed handwashing place | 84 | 78 | 3 (3.8) | 1 |  |
| Mobile handwashing place | 180 | 169 | 8 (4.7) | 1.5 (0.4 – 6.3) | 0.595 |
| No handwashing place | 321 | 293 | 15 (5.1) | 1.4 (0.4 – 5.5) | 0.605 |
| Presence of soap in the handwashing area | 139 | 130 | 7 (5.4) | 1.2 (0.4 – 3.1) | 0.744 |
| Lack of soap in the handwashing area | 446 | 410 | 19 (4.6) | 1 |  |
| Safe drinking water sources | 72 | 65 | 4 (6.2) | 1.17 (0.4 –3.9) | 0.792 |
| Basic drinking water sources | 513 | 475 | 22 (6.7) | 1 |  |
| Safe cooking water sources | 80 | 72 | 4 (5.6) | 1.04 (0.3 – 3.4) | 0.945 |
| Basic cooking water sources | 505 | 468 | 22 (4.7) | 1 |  |
| Drinking water treated | 277 | 255 | 9 (3.5) | 0.6(0.2 – 1.3) | 0.190 |
| Drinking water not treated | 308 | 285 | 17 (6.0) | 1 |  |
| Cooking water treated | 77 | 72 | 2 (2.8) | 0.5(0.1 – 2.3) | 0.360 |
| Cooking water not treated | 508 | 468 | 24 (5.1) | 1 |  |
| Food reheated to boiling | 126 | 114 | 5 (4.4) | 1.1 (0.4 – 3.1) | 0.889 |
| Food not reheated to boiling | 452 | 420 | 21 (5.0) | 1 |  |
| Milk reheated to boiling | 174 | 159 | 6 (3.8) | 1.5 (0.5 –3.9) | 0.460 |
| Milk not reheated to boiling | 392 | 363 | 20 (5.5) | 1 |  |
| Handwashing before food preparation | 219 | 207 | 13 (6.3) | 1.7 (0.7 –3.8) | 0.233 |
| No handwashing before food preparation | 366 | 333 | 13 (3.9) | 1 |  |
| Handwashing before feeding the  child | 305 | 285 | 16 (5.6) | 1.4 (0.6 –3.3) | 0.455 |
| No handwashing before feeding the  child | 280 | 255 | 10 (3.9) | 1 |  |
| Rodents observed | 370 | 341 | 18 (5.3) | 1.4 (0.6 – 3.4) | 0.482 |
| No rodents observed | 215 | 199 | 8 (4.0) | 1 |  |
| Animal feces in the compound | 42 | 36 | 5 (13.9) | 4.4 (1.4 –14.3) | 0.013* |
| No animal feces in the compound | 543 | 504 | 21 (4.2) | 1 |  |
| Human feces in the compound | 14 | 14 | 1 (7.1) | 1.6 (0.2 – 14.1) | 0.671 |
| No human feces in the compound | 571 | 526 | 25 (4.8) | 1 |  |
| Garbage in compound | 197 | 183 | 12 (6.6) | 1.9 (0.8 – 4.5) | 0.165 |
| No garbage in  compound | 388 | 357 | 14 (3.9) | 1 |  |
| **Food consumption** |  |  |  |  |  |
| Consumed eggs | 65 | 57 | 3 (5.3) | 1.2(0.3 – 4.2) | 0.834 |
| Did not consume eggs | 520 | 483 | 23 (4.8) | 1 |  |
| Consumed cow’s milk | 350 | 324 | 22 (6.8) | 3.9 (1.3 – 11.8) | 0.016* |
| Did not consume cow’s milk | 235 | 216 | 6 (2.8) | 1 |  |
| Consumed packet milk | 122 | 113 | 5 (4.4) | 0.9 (0.3 – 2.7) | 0.920 |
| Did not consume packet milk | 463 | 427 | 21 (4.9) | 1 |  |
| Consumed yogurt | 103 | 92 | 4 (4.3) | 0.88 (0.3 – 2.7) | 0.828 |
| Did not consume yogurt | 482 | 448 | 22 (4.9) | 1 |  |
| Drank tea | 291 | 271 | 16 (5.9) | 1.7 (0.7 – 3.9) | 0.236 |
| Did not drink tea | 294 | 269 | 10 (3.7) | 1 |  |
| Consumed broth | 86 | 83 | 6 (7.2) | 1.7 (0.6 – 4.9) | 0.343 |
| Did not consume broth | 499 | 457 | 20 (4.4) | 1 |  |
| Consumed homemade porridge | 299 | 278 | 12 (4.3) | 0.8 (0.4 – 1.8) | 0.602 |
| Did not consume homemade porridge | 286 | 262 | 14 (5.3) | 1 |  |
| Consumed vegetables | 314 | 285 | 15 (5.3) | 1.2 (0.5 – 2.6) | 0.669 |
| Did not consume vegetables | 271 | 255 | 11 (4.3) | 1 |  |
| Ate meat | 112 | 99 | 3 (3.0) | 0.5 (0.1 – 1.9) | 0.309 |
| Did not eat meat | 473 | 441 | 23 (5.2) | 1 |  |
| **Animal ownership** |  |  |  |  |  |
| Chicken ownership  Yes | 101 | 95 | 10 (10.5) | 3.0 (1.3 – 7.1) | 0.013* |
| Chicken ownership  No | 484 | 445 | 16 (3.6) | 1 |  |
| Pig ownership Yes | 20 | 19 | 1 (5.3) | 0.8(0.1 – 7.2) | 0.823 |
| Pig ownership no | 570 | 522 | 25 (4.8) | 1 |  |
| Cattle ownership  Yes | 16 | 15 | 2 (13.3) | 2.6 (0.5 – 14.2) | 0.270 |
| Cattle ownership  No | 569 | 525 | 24 (4.6) | 1 |  |
| Goat ownership  Yes | 14 | 14 | 3 (21.4) | 5.4 (1.2 – 24.3) | 0.029* |
| Goat ownership No | 571 | 526 | 23 (4.4) | 1 |  |
| Sheep ownership  Yes | 6 | 6 | 1 (16.7) | 3.3 (0.3 –37.0) | 0.327 |
| Sheep ownership  No | 579 | 534 | 25 (4.7) | 1 |  |
| Dog ownership Yes | 36 | 34 | 4 (11.8) | 2.57 (0.8 – 8.8) | 0.132 |
| Dog ownership No | 549 | 506 | 22 (4.3) | 1 |  |
| Cat ownership Yes | 98 | 91 | 5 (5.5) | 1.1 (0.4 – 3.1) | 0.888 |
| Cat ownership No | 487 | 449 | 21 (4.7) | 1 |  |

* p-value<0.05
